# Heterologous expression of plasmodial proteins for structural studies and functional annotation

**DOI:** 10.1186/1475-2875-7-197

**Published:** 2008-10-01

**Authors:** Lyn-Marie Birkholtz, Gregory Blatch, Theresa L Coetzer, Heinrich C Hoppe, Esmaré Human, Elizabeth J Morris, Zoleka Ngcete, Lyndon Oldfield, Robyn Roth, Addmore Shonhai, Linda Stephens, Abraham I Louw

**Affiliations:** 1 Department of Biochemistry, University of Pretoria, Pretoria, South Africa; 2 Department of Biochemistry, Microbiology & Biotechnology, Rhodes University, Grahamstown, South Africa; 3 Department of Molecular Medicine and Haematology, University of the Witwatersrand/National Health Laboratory Service (NHLS), Johannesburg, South Africa; 4 Council for Scientific and Industrial Research, CSIR Biosciences, Pretoria, South Africa; 5 African Centre for Gene Technologies (Joint initiative of the CSIR, the University of Pretoria and the University of the Witwatersrand), Pretoria, South Africa; 6 Department of Biochemistry and Microbiology, Zululand University, Kwadlangezwa, South Africa

## Abstract

Malaria remains the world's most devastating tropical infectious disease with as many as 40% of the world population living in risk areas. The widespread resistance of *Plasmodium *parasites to the cost-effective chloroquine and antifolates has forced the introduction of more costly drug combinations, such as Coartem^®^. In the absence of a vaccine in the foreseeable future, one strategy to address the growing malaria problem is to identify and characterize new and durable antimalarial drug targets, the majority of which are parasite proteins. Biochemical and structure-activity analysis of these proteins is ultimately essential in the characterization of such targets but requires large amounts of functional protein. Even though heterologous protein production has now become a relatively routine endeavour for most proteins of diverse origins, the functional expression of soluble plasmodial proteins is highly problematic and slows the progress of antimalarial drug target discovery. Here the *status quo *of heterologous production of plasmodial proteins is presented, constraints are highlighted and alternative strategies and hosts for functional expression and annotation of plasmodial proteins are reviewed.

## Background

Malaria is a devastating disease and its long-term control and eradication are still a long way off. There is no licensed vaccine and our dispensary of affordable, effective drugs is in danger of being depleted in the short term owing to increased drug resistance. The only solution currently is to resort to drug combinations coupled to insecticide spraying and/or the distribution of insecticide-treated bed nets. A number of novel drug targets have been identified and/or validated, and are being pursued for the development of new drugs. However, it is obvious from the nearly 60% of hypothetical proteins in the parasite genome that have not yet been annotated, that relatively little of the biology of the parasite is known compared to the biology of for example, humans, yeast or plants [1]. The potential for the discovery of new and better drug targets is thus considerable. Bioinformatics tools are useful in characterizing specific properties of selected proteins and can even suggest possible functions of hypothetical proteins. However, protein function and structural properties can only be confidently inferred from biochemical and cell biological experimentation, whilst the design of inhibitors and evaluation of their interaction with the protein of interest are ultimately dependent on the availability of soluble and functional proteins. It is widely accepted that a specific protein of interest can only in rare instances be isolated in sufficient quantities from the natural host cell for downstream studies. Heterologous expression of the selected protein is therefore of paramount importance. This review highlights the properties that have been associated with difficult-to-express plasmodial proteins and provides insights into the variety of options available for the heterologous expression of malaria parasite proteins for structural and functional annotation studies.

### *Escherichia coli *as workhorse for plasmodial protein expression

*Escherichia coli *is still the preferred host for the heterologous expression of recombinant proteins due to cost considerations, speed, ease of use and genetic manipulation. However, for many proteins [2], including plasmodial proteins, this approach is fraught with difficulties as reflected by the paucity of plasmodial protein structures in the Protein Data Bank (PDB, ~250), when compared to the approximately 50,000 protein structures available in the PDB. One of the major limitations of this system is the expression of insoluble recombinant proteins sequestered in inclusion bodies [3,4], as evidenced by the results from several large scale protein expression endeavours [5-7]. The Structural Genomics of Pathogenic Protozoa group (SGPP, University of Washington), selected 1,000 *Plasmodium falciparum *open reading frames for expression from a modified pET vector in *E. coli *BL21 Star cells [5]. Of these, expression was obtained for approximately one third with only 63 proteins expressed in soluble form. Another large scale attempt at expressing plasmodial proteins yielded only nine soluble, purified proteins out of 95 [6]. Lastly, the Structural Genomics Consortium (SGC, Toronto, Canada) selected 400 distinct *P. falciparum *target genes representing different cellular classes, along with orthologues from four other *Plasmodium *species as well as *Cryptosporidium parvum *and *Toxoplasma gondii *[7]. From the selected 1008 genes, 30% produced soluble proteins from pET vectors and *E. coli *BL21 CodonPlus (DE3)-RIL cells of which ~100 crystallized, culminating in 36 crystal structures. The most significant conclusion from this study was that the percentage of pure protein, crystals and structures could be doubled when orthologues from all six genomes were included compared to those obtained from *P. falciparum *alone.

The *E. coli *host expression system can be optimized to ensure expression plasmid stability, the absence of harmful natural proteases and availability of the relevant genetic elements, e.g. DE3 [8]. The most popular expression vector/*E. coli *strain combination seems to be based on pET vectors and the BL21 (DE3) *E. coli *strain as evidenced by the experiences of structural genomics consortia with the expression and purification of more than 100 000 proteins from Eubacteria, Archea and Eukarya. These include the *recA *negative strains (BLR strain) for the stabilization of plasmids containing repetitive sequences, *lacY *mutants (Tuner series), which enable adjustable levels of protein expression and the *trzb*/*gor *negative mutants (Origami) for improved cytoplasmic disulphide bond formation [8]. Gene expression is also controlled by the decay rate of mRNA and host strains that are deficient in specific RNases may enhance expression by protecting the mRNA from degradation [8]. In addition, several counter strategies can be employed to overcome codon bias and to assist disulphide bond formation, protein phosphorylation and protein refolding (reviewed in [9]). Furthermore, fusion tags such as hexahistidine, Glutathione-S-transferase (GST) and others (reviewed in [10]), often improve solubility of the recombinant protein and their effect can be predicted bioinformatically, which is useful to guide the investigator in the selection of an appropriate vector. New technologies include the Gateway System, which uses the *araBAD *promoter designed for tight control of background expression and L-arabinose-dependent graded expression of the target protein. It also allows flexibility in terms of a rapid and highly efficient route to multiple expression and functional analysis options and has been successfully applied for high-throughput recombinatorial cloning of *P. falciparum *functional molecules (especially vaccine candidates) [6] and as a transfection vector set [11].

Overexpression of proteins in *E. coli *frequently leads to insoluble protein in inclusion bodies due to metabolic stress and it is worthwhile to explore various expression conditions such as lower incubation temperatures and enriched media to promote expression of soluble recombinant proteins [9,12,13]. Interesting new advances, which have thus far not been applied to plasmodial protein expression include the use of bacterial bioreactors utilizing a combination of the above-mentioned attributes, as well as low temperature, and the induction of an mRNA-specific endoribonuclease causing host cell growth arrest and culture condensation. These bioreactors facilitate expression of heterologous proteins to ~30% of total cellular protein in a stable, condensed fashion without toxic effects due to the arrested state of the *E. coli *[14]. Recent experiments indicated that in response to expression of insoluble proteins, direct targets of the σ^32 ^heat shock sigma factor are highly expressed [15,16]. Manipulation of this functional response could provide a wide-ranging mechanism for improving recombinant protein solubility. Thus the σ^32^-regulon would be induced before the accumulation of insoluble proteins, producing a larger pool of chaperones and a simultaneous increase in protein folding [15].

Protein characteristics that jeopardized soluble expression of recombinant plasmodial proteins were found to include higher molecular weight (> 56 kDa), greater protein disorder, more basic pI (> 6) and lack of homology to *E. coli *proteins. In addition, the presence of low complexity regions (SEG > 29%) and *Plasmodium-*specific inserts, transmembrane regions, as well as signal peptides, transit peptides and export motifs, have a negative impact on protein expression [5-7]. The presence of conserved sequences for prokaryotic promoters and ribosome binding sites within the plasmodial gene of interest, as well as unusual mRNA and/or stable secondary structures should also be avoided [17]. The difficulties of expressing plasmodial proteins in *E. coli *have also been attributed to toxicity of plasmodial proteins [7] and the difference in amino acid content between proteins of the two species, which imposes a metabolic burden on the bacterial host. Ribosome stalling is triggered by the limited availability of specific amino acids, which causes errors in and/or truncated forms of the recombinant protein. The prevalence of codons rare in *E. coli*, especially Arg codons (AGA and AGG), can also cause the ribosome to pause and bring about the early termination of expression due to tRNA exhaustion [18]. The marked A+T bias in the *P. falciparum *genome [19,20] has also been implicated in low level expression of proteins, although two large scale studies revealed that A+T richness *per se *does not appear to affect expression [5-7].

It is clear that the ability to predict soluble protein expression is limited, but bioinformatics analyses can guide the design of strategies to deal with features associated with difficult-to-express proteins in *E. coli*. Proteins of the apicomplexan species have unusually high contents of long, disordered regions compared to other eukaryotes. In addition, the mammalian malaria parasites are particularly enriched in intrinsically unstructured proteins when compared to those of the rodent species [21]. Intrinsically unstructured and low complexity regions overlap with each other to some extent but often coexist discretely in the *P. falciparum *genome [22]. These regions, their extraordinary lengths as well as the occurrence of bifunctional plasmodial proteins expressed from one open-reading frame, impose significant constraints on the probability of obtaining soluble expression of these proteins [19,20,23]. The intrinsically disordered regions in proteins have distinct functions [24] and their complete removal is not necessarily a viable option even if it occurs at the N- or C-terminal ends of proteins [25] without prior consideration to functional motifs that can be identified by bioinformatics methods and retained in gene constructs [26]. Alternatively, systematic N- and C-terminal deletions can be conducted to promote soluble expression [27]. It is also worthwhile taking note of the application of isolation methods specifically designed for isolation of proteins with high structural disorder in soluble form [28,29] and newer methods for co-expression of proteins with interacting protein partners to improve soluble expression of largely unstructured proteins [30-32].

Beyond the obvious removal of e.g. signal peptides and/or minor genetic engineering of other interfering traits [8,19,20,23] other options include searching for orthologous genes in other plasmodial species and apicomplexan parasites with attributes more amenable to soluble expression (see e.g. [23]) or expression of single domains of bifunctional or unstructured proteins [33-35].

Faced with the myriad of possibilities described above it is gratifying to note that newer methods are available that are especially applicable in academic laboratories for the efficient parallel screening of multiple constructs with minimal cost. These methods include novel expression vectors, micro-expression in 96-well format, a colony filtration blot procedure and new shakers or fermentors allowing for the simultaneous expression analyses of several different protein samples [13].

A survey was performed on the expression of potential antimalarial drug targets from the WHO TDR Targets database [36]. From the 57 potential drug targets with druggability evidence indices above 0.7, 32 were chosen. Hypothetical or putative proteins were excluded (Table 1). From these, no evidence for the expression of 19% were obtained whereas all of the remaining proteins could be expressed in *E. coli*, one in yeast and two proteins in cell-free systems. His-tagging for isolation of the protein had preference over other tags. Special conditions utilized to express these plasmodial proteins include truncations, codon optimization and tRNA additions. The following sections introduce alternative strategies and expression hosts worth exploiting.

**Table 1:** Heterologous expression of 32 potential antimalarial drug targets.

**PlasmoDB annotation**	**Product**	**Drug-ability Index (max is 1)**	**MM (Da)**	**pI**	**Heterologous Expression (Ref)**	**Tag**	**Special conditions (e.g. codon adaptation, TM, trunctions)**	**Structure (PDB code)**
PF14_0053	Ribonucleotide reductase small subunit	1	40600	5.2	*E. coli *[212]	His and GST	-	-
PFD0830w	Bifunctional dihydrofolate reductase-thymidylate synthase	1	71738	7.2	- *E. coli *(various) - *S. cerevisiae *(complemeted) (various) - Cell free systems [207]	-	- Purposely truncated versions or domains - Codon optimised - *S. cerevisiae *adapted	1J3I 1J3J 1J3K
PFE0520c	Topoisomerase I	1	98110	9.8	-	-	-	-
PFF0160c	Dihydroorotate dehydrogenase, mitochondrial precursor	1	65559	9.4	*E. coli *[213]	His	N-terminally truncated pRIL tRNA additions	1TV5
PFI1020c	Inosine-5'-monophosphate dehydrogenase	1	56151	8.0	-	-	-	-
PF11_0377	Casein kinase 1	0.9	37631	9.7	*E. coli *[214]	His		1lhx
PF14_0192	Glutathione reductase	0.9	56679	8.1	*E. coli *complementation [215]	His	Gene complementation	1ONF
PFI1685w	cAMP-dependent protein kinase catalytic subunit	0.9	40197	9.1	-	-	-	-
PFI1170c	Thioredoxin reductase	0.9	59687	7.8	*E. coli *[216]	His	-	-
MAL13P1.279	Protein kinase 5	0.9	32997	8.0	*E. coli *[217]	His	pRIL tRNA addition	1OB3
PFC0525c	Glycogen synthase kinase 3	0.9	51616	5.2	*E. coli *[218]	V5/His-/thioredoxin	-	-
PFL2250c	Rac-beta serine/threonine kinase	0.9	88096	10	*E. coli *[219]	His or GST	pRIL tRNA addition	-
PF10_0121	Hypoxanthine phosphoribosyltransferase	0.8	70076	4.6	*E. coli *[220]	-	-	1CJB
PF10_0165	DNA polymerase delta catalytic subunit	0.8	127072	8.7	*E. coli *[221]	3-galactosidase	-	-
PF10_0322	S-adenosylmethionine decarboxylase-ornithine decarboxylase	0.8	168171	6.3	*E. coli *[222]	STREP	-	-
PF13_0141	L-lactate dehydrogenase	0.8	34108	7.6	*E. coli *[223,224]	**-**	- 1 TM/1 signal peptide - 2xYT - reducing cell growth temperature to 15°C	1CEQ
PF13_0287	Adenylosuccinate synthetase	0.8	50066	7.7	*E. coli *[225]	-	Low-temperature inductions were carried out at 20°C for 12 h	1P9B
PFI1090w	S-adenosylmethionine synthetase	0.8	44844	6.7	*E. coli *[226]	His		-
PF14_0076	Plasmepsin 1 precursor	0.8	51461	7.2			1 TM	1PFZ
PF14_0077	Plasmepsin 2 precursor	0.8	51481	5.3	*E. coli *[227]		- 1 TM - Solubilised and refolded	1SME
PF14_0125	Deoxyhypusine synthase	0.8	57429	6	*P. vivax *expressed		1 TM	-
PF14_0127	N-myristoyltransferase	0.8	47971	8.3	*E. coli *[228]	His	-	-.
PF07_0029	Heat shock protein 86	0.8	86167	4.7	*E. coli *[229]	His	-	-
PF14_0164	NADP-specific glutamate dehydrogenase	0.8	52547	7.3	*E. coli *[230,231]	-	-	2bma
PF14_0378	Triose-phosphate isomerase	0.8	27935	6.4	*E. coli *[232]	-	-	1lyx
PFD0590c	DNA polymerase alpha	0.8	225404	8.4	-	-	-	-
PFE1050w	Adenosylhomocysteinase(S-adenosyl-L-homocystein e hydrolase)	0.8	52840	5.7	*E. coli *[233,234]	-	-	1v8b
PFF0730c	Enoyl-acyl carrier reductase	0.8	49763	9.5	*E. coli *[235]	MBP	pRIL tRNA additions	1nhg
PFI1260c	Histone deacetylase	0.8	51376	6.3	Cell free systems [236]	No	-	-
PFI1105w	Phosphoglycerate kinase	0.8	45427	8.1	*E. coli *[237]	His	-	1lgi
PFD1050w	Alpha-tubulin II	0.8	49878	4.6	-	-	-	-
PFD0420c	Flap exonuclease 1	0.8	76868	8.1	-	-	-	-

The TDR Targets database (tdrtargets.org) were searched for *P. falciparum *genes with a druggability evidence index ≥0.7. Hypothetical or putative proteins were excluded.

### Codon-optimization vs -harmonization of plasmodial proteins

Codon bias refers to the high frequency preferential use of a particular codon coding for an amino acid within an organism. There is a high level of codon mismatch between *P. falciparum *and expression hosts, such as *E. coli *and two different strategies have been used to minimize codon bias. Firstly, the intracellular tRNA pool can be expanded by using plasmids which encode rare tRNAs used in certain organisms, for example *E. coli *[23]. Secondly, altering the codons of the target genes to suit the codon preference of the non-natural expression host is a process referred to as 'codon-optimization' [18,37-39]. This approach has met with some success, especially in antimalarial vaccine targets [17,18,40,41], but unfortunately the expression of several other malaria proteins was not improved through codon-optimization alone [42]. As an example, SGPP [5] reported that nine out of the twelve *P. falciparum *genes that had been synthesized with optimized codons could still not be expressed in *E. coli*, and the three proteins that were expressed were in inclusion bodies. This led them to conclude that codon usage did not have a significant impact on protein expression.

During translation, ribosomes often encounter low frequency codons that may cause translational pausing. It is the stalling at these essential pause sites that halts protein expression and allows a predetermined interval which permits correct folding of the protein [38,43]. The technique of codon-harmonization or translational attenuation refers to the process of recognizing the low usage frequency codons within the natural host (*P. falciparum*) and altering the code of the gene to be expressed to codons recognized as low usage frequency codons by the non-natural host [38,39]. These changes ensure the positional codon frequency of low/intermediate and high usage codons to remain the same in the non-natural host, which allows for the translational processes to match that of the natural host. By allowing the translational machinery to move and pause at the correct sites, the folding of the secondary and tertiary structures occur as in the natural host [39,43-45]. This approach has been remarkably successful in the heterologous production of a liver-stage antigen of *P. falciparum *(LSA-1) previously refractory to recombinant expression even with codon-optimization as well as co-expression with a rare frequency tRNA plasmid [45].

### Molecular chaperones to aid plasmodial protein expression

One of the difficulties in heterologous expression of plasmodial proteins is incorrect folding of the proteins in the non-natural host. Molecular chaperones of the heat shock protein family (Hsp) ensure that proteins in a cell are properly folded and functional (reviewed in [46,47]). Of particular interest is the demonstration that the co-transformation of target genes and chaperones from the same species cloned into different plasmids resulted in an increased yield of soluble protein in *E. coli *[32,48].

Hsp70, a 70 kDa heat shock protein, is an important class of molecular chaperones involved in protein folding by ATP-controlled cycles of substrate binding and release. At least six genes for Hsp70-type proteins have been identified in *P. falciparum *[49,50] and *Pf*Hsp70-1 has features to justify its candidature as co-expression partner, including successful overexpression in *E. coli *[51]. *Pf*Hsp70-1 was able to reverse the thermosensitivity of *E. coli *cells in an *in vivo *assay, which suggests that it interacted with *E. coli *protein substrates, protecting them against heat stress [50,52]. *Pf*Hsp70-1 is able to recognize typical Hsp70 peptide substrates [53], due to its ability to suppress protein aggregation *in vitro *[54]. A study of the interactome of PfHsp70-1 showed that it potentially recognizes a wide range of *P. falciparum *substrates, including apicoplast proteins [50,55,56] and mutating these residues on an apicoplast transit peptide led to the protein being mistargeted [57].

Like other Hsp70 proteins, *Pf*Hsp70-1 needs an Hsp40 co-chaperone partner to function as a refoldase, otherwise its role during co-expression with a target protein in *E. coli *would be a mere suppression of protein aggregation. *Pf*Hsp70 and *Pf*Hsp40 have been identified in the parasitophorous vacuole and Maurer's clefts, implicating them in erythrocyte quality control of parasitic proteins and their possible export into the erythrocyte [49,58,59]. More than forty Hsp40 proteins have been identified and the *Pf*Hsp40 for *Pf*Hsp70 is predicted to be PF14_0359 [56,60]. It is therefore conceivable that molecular chaperones from *P. falciparum *could be harnessed as co-expression partners during the overproduction of target proteins in *E. coli *to provide a specialized folding pathway.

### Refolding of incorrectly folded, insoluble plasmodial proteins

The immense drawback during high level protein expression in *E. coli *is the production of insoluble proteins in inclusion bodies [4,61]. Due to the reversibility of protein aggregation in bacteria, several strategies are employed to isolate and refold inclusion bodies since they are a good source of reasonably pure polypeptides, which could provide protein in the native conformation after refolding [62]. Inclusion bodies almost exclusively contain the over-expressed recombinant protein and very little host protein, ribosomal components or DNA/RNA fragments [63]. Protein refolding is a complicated procedure requiring several optimization steps [64]. Solubilization of inclusion bodies results in soluble protein in its non-native conformation, which needs to be refolded [64]. The inclusion of chaotropic reagents ensures the successful formation of the native structure [64,65] by allowing intra-molecular interactions and/or preventing inter-molecular interactions.

Sirawaraporn *et al *showed that disaggregated and refolded *P. falciparum *dihydrofolate reductase (DHFR) from inclusion bodies yielded active protein in sufficient amounts for enzyme function determinations [34]. Optimal refolding of *P. falciparum *cysteine protease falcipain-1 resulted in an enzymatically active monomeric form of this protein, which exhibited high immunogenic activity [66]. Refolding procedures were also used to obtain high levels of *P. falciparum *cysteine protease falcipain-2, resulting in the crystallization of this protein [67]. Immunologically active *P. falciparum *42 kDa MSP1_42 _was obtained by refolding of MSP1_42 _as a fusion protein from inclusion bodies [68]. Due to the undesirability of fusion tags in clinical trials, refolding protocols were utilized to obtain the first recombinant form of *P. falciparum *MSP1_42 _without a 6× His affinity tag [69].

Extensive optimization is required to produce functional refolded plasmodial proteins, but commercial kits are now available, and refolding represents a valuable, under-utilized tool to improve the yield of soluble recombinant *P. falciparum *proteins.

### Alternative systems for expression of plasmodial proteins

#### Yeasts

The commonly-used yeast species *Saccharomyces cerevisiae *and *Pichia pastoris *have been exploited extensively for the heterologous expression of plasmodial proteins, both for preparative purposes and functional characterization. *S. cerevisiae *is a popular choice as host owing to the ease with which it can be manipulated genetically and to the extraordinary amount of information accumulated about its molecular biology and physiology [70]. The use of this model organism varies from vaccine production [71] to functional characterization, drug discovery and resistance studies and elucidating parasite protein interaction networks ("interactomes") through high-throughput 2-hybrid analyses (e.g. [55,72]).

Expression in *S. cerevisiae *is typically controlled by inducible promoters, e.g. ADH2 (a glucose-repressed alcohol dehydrogenase promoter), while N-terminal fusion to a pheromone results in efficient secretion of recombinant protein from the yeast cells [73,74]. Coupled with carefully controlled growth in fermenters, impressive yields of 10 – 75 mg/L have been reported [75,76]. Secretion into the culture medium represents a major advantage of the yeast system over *E. coli*, as it circumvents formation of inclusion body precipitates and simplifies subsequent purification. Furthermore, trafficking through the yeast secretory compartments facilitates appropriate disulphide bond formation and protein folding, which is especially important for malaria vaccine production [76-78]. However, the latter advantage may have a sting in its tail: disulphide bonding in individual proteins may be heterogeneous, resulting in a population of structural conformers, not all of which contain the conformationally correct epitopes [76]. In addition, the yeast secretory pathway may result in spurious O- and N-linked glycosylation of the heterologous proteins [79]. The latter is especially relevant for plasmodial proteins, since these are rarely (if ever) glycosylated in their native state. The problem may be circumvented to some extent by altering the coding sequence to remove potential N-glycosylation motifs or inclusion of N-glycosylation inhibitors [80]. Limitations in yeast systems that can reduce the final yield include the codon usage of the expressed gene, the gene copy number, efficient transcription by using strong promoters leading to the depletion of precursors and energy, translocation, processing and folding in the endoplasmic reticulum and Golgi, as well as protein turnover by proteolysis [81]. Cells which produce high levels of proteins can accumulate unfolded protein in the ER, which aggregates, overwhelms and eventually shuts down the secretory pathway [82]. The yeast proteins that assist in folding and disulphide bond formation differ from their counterparts in higher eukaryotes, which may affect folding of foreign proteins [83]. *Saccharomyces cerevisiae *moreover recognizes distinct A+T containing codons as termination signals which could lead to the truncation of encoded proteins especially of the *P. falciparum *genome. Due to this codon incompatibility, synthetic genes incorporating a yeast codon bias are often used [84].

Recombinant vaccine production in *S. cerevisiae *has focused largely on the preparation of transmission-blocking vaccines [76]. The principal components of these experimental vaccines that block transmission of malaria parasites to *Anopheles *are the epidermal growth factor (EGF)-like domains of *Pfs*25 (of *P. falciparum*) or *Pvs*25 (of *P. vivax*), a glycosylphosphatidyl inositol (GPI)-anchored surface protein of the mosquito-stage zygotes and ookinetes of the parasite [85-87]. Additional vaccine expression studies in *S. cerevisiae *have centred on the C-terminal domain of the major GPI-anchored surface protein of merozoites, merozoite surface protein 1 (MSP1_19_) [88,89]. Despite these promising efforts, *S. cerevisiae *has largely been superseded by *Pichia pastoris *as the host cell of choice for vaccine production (see discussion below). Nonetheless, *S. cerevisiae *has remained relevant to malaria research through its highly characterized biology and amenability to genetic manipulation, which allows it to be exploited for functional characterization through complementation experiments. In these studies, the ability of the heterologously expressed plasmodial proteins to rectify or modify aberrant phenotypes of conditional yeast deletion mutants is typically assessed. The studies are aided by the fact that relevant yeast mutants may be obtained from extensive repositories e.g. EUROSCARF [90], the *Saccharomyces *Genome Deletion Project [91] and the ATCC [92]. Often, the plasmodial coding sequence is modified to increase G+C content and improve yeast codon compatibility, single-copy yeast episomal plasmids are used to keep heterologous protein expression to physiologically relevant levels and expression is driven by constitutive (e.g. ADH1) or inducible (e.g. GAL1) promoters. Several classes of plasmodial proteins have been characterized in this fashion including integral membrane transporters [93-95]; phospholipid synthesis and modifying enzymes [96-99]; cell cycle, transcriptional and translational regulators [100-103]; and possible drug targets e.g. Vitamin B6 synthesis enzymes (*Pf*Pdx1 and *Pf*Pdx2, [104]) and folate metabolism proteins, dihydrofolate reductase-thymidylate synthase, (DHFR-TS) and dihydrofolate synthase-folylpolyglutamate synthase (DHFS-FPGS, [105]). A highly informative series of experiments focused on DHFR-TS demonstrated how yeast complementation may be used as a versatile alternative to preparing soluble purified protein for the characterization and exploitation of parasite drug targets. The complementation system was employed to determine the effects of DHFR mutations in parasite field isolates on sensitivity/resistance to antifolate drugs [106,107], the contribution of various DHFR mutations to antifolate resistance and predict future resistance trends by using a PCR-based random mutagenesis approach [108], and to screen novel antifolate compounds for activity against sensitive and resistant alleles of DHFR [109,110].

Lastly, *S. cerevisiae *is widely used to determine protein binding partners by yeast two-hybrid analysis which has resulted in a characterization of the *P. falciparum *interactome and identification of a network of 2,846 interactions between 1,312 plasmodial proteins which have minimal similarity to interaction networks of other model organisms [55,72].

Some of the non-conventional yeasts, such as *Kluyveromyces lactis *and the methylotrophs *Pichia pastoris *and *Hansenula polymorpha *(now known as *Pichia angusta*, [111]) have also been developed as expression systems. *Pichia pastoris *is now the most frequently used yeast species for heterologous protein expression in general. It shares the underlying features and methodology of *S. cerevisiae*, with one notable exception: its biology is considerably less well characterized. Hence, it is used almost exclusively for preparative expression as opposed to the large number of functional studies that have been carried out in *S. cerevisiae*. However, the same principles apply: plasmodial sequences are altered to incorporate a yeast codon bias; potential N-glycosylation sites in the plasmodial sequences are removed by mutation; expression is driven by an inducible promoter (the methanol-inducible alcohol oxidase promoter, AOX1); efficient secretion into the culture medium is afforded by N-terminal fusion to the *S. cerevisiae *prepro-α-factor leader peptide and C-terminal tags are incorporated to facilitate purification from the medium. The redox environment and co-factors in the yeast secretory pathway should enhance correct folding, solubility and intra-molecular disulphide bonding of vaccine proteins that rely critically on conformational epitopes to generate a protective immune response; the latter may be compromized by spurious and unpredictable O-glycosylation of the heterologous protein, as well as the formation of disulphide bond conformers with reduced protective immunogenicity [112,113]. It appears that the use of *Pichia pastoris *may improve heterologous protein yield, compared with *S. cerevisiae*, and levels of 30 mg/L to more than 1 g/L of purified protein have consistently been reported, especially when combined with batch-fed fermentation [113-121]. In addition, the ratio of desirable/undesirable conformers may be improved in *Pichia pastoris *[113]. In one study, this was further enhanced by co-expressing the heterologous protein with *P. falciparum *or *Pichia pastoris *protein disulphide isomerase (PDI), which improved yield as well as reducing O-glycosylation [121]. Exoprotease truncation of heterologous proteins may also be encountered in *Pichia pastoris*, but was circumvented in one case by alteration of the N-terminal sequence of a *P. falciparum *circumsporozoite protein (CSP)-derived vaccine product prone to digestion [122].

A number of plasmodial vaccine proteins have been successfully expressed in *Pichia pastoris*, notably merozoite proteins required for erythrocyte invasion (AMA-1, EBA-175, MSP-1, MSP-3), the sporozoite-stage antigen CSP, and transmission-blocking mosquito-stage antigens (*Pfs*25) [41,112-120,123-127]. A notable exception to the vaccine preparation studies in which *Pichia pastoris *has been used almost exclusively in malaria research is the functional characterization of *P. falciparum *transporters thought to be involved in quinoline drug resistance, *Pf*CRT (*P. falciparum *chloroquine resistance transporter) and a P-glycoprotein homologue, Pgh-1 [128-131]. In addition to optimizing the codon usage for yeast expression, the plasmodial sequences were altered to remove poly(A) stretches, potential yeast-specific premature termination signals and predicted stem-loop structures. A 5' Kozak sequence was added, as well as C-terminal fusions to bacterial biotin acceptor domains to enable subsequent detection and/or purification. Successful membrane-localized expression of the transporters was followed by functional characterization of these proteins.

#### Baculovirus

The use of baculovirus-infected insect cells to express recombinant proteins is well established [132]. A lytic virus, *Autographa californica *nuclear polyhedrosis virus, is most commonly used. The gene of interest is cloned into a transfer vector, which is co-transfected with viral DNA into *Spodoptera frugiperda *(usually *Sf*9 or *Sf*21) insect cells. In a homologous recombination event, the foreign gene, under the control of a strong viral promoter such as polh or p10, is integrated into the viral genome and recombinant protein is produced in the infected cells. A C-terminal or N-terminal fusion tag is incorporated into the protein to facilitate purification. The large size of the viral genome makes it amenable to the insertion of large segments of foreign DNA. Numerous baculovirus systems, including a variety of transfer vectors, are now commercially available and technological improvements have simplified the production and isolation of recombinant baculovirus.

This approach has been successfully applied to several *P. falciparum *proteins. In one large study, 17 proteins that were expressed as insoluble inclusion bodies in *E. coli *were transferred to a baculovirus/*Sf*21 system, which resulted in high levels of soluble protein in one case (11.7 mg/500 ml culture) and lower levels of six other proteins, ranging from 0.6 to 7.2 mg/500 ml culture [5]. The low yield can easily be overcome by scaling up the procedure.

The baculovirus system uses eukaryotic insect cells to express recombinant proteins and thus the folding and assembly of newly synthesized polypeptides may be similar to the natural process in *Plasmodium*. As a result, soluble recombinant baculovirus *P. falciparum *proteins are likely to fold correctly to produce immunologically active proteins that are recognized by conformation-specific monoclonal antibodies. This feature is vital when assessing the immunogenic response of the host to a recombinant *Plasmodium *protein that is considered to be a vaccine target and several studies on *P. falciparum *antigens illustrate this aspect. Recombinant domains of merozoite surface protein MSP-1 [133,134], CSP [135,136] and a erythrocyte membrane protein *Pf*EMP1 variant [137] retained their native conformational epitopes and elicited antibody responses. In contrast, recombinant *Pf*MSP-1 expressed in yeast had a different conformation to the native protein and was immunologically inactive [133]. Erythrocyte binding antigen (*Pf*EBA-175) has also been expressed in a baculovirus system in soluble form that was functionally active [138,139]. Additional examples are apical membrane antigen (*Pf*AMA-1), which was successfully purified from baculovirus-infected insect cells [140] and recombinant serine repeat antigen (*Pf*SERA), which was subsequently processed into several fragments that reflect the *in vivo *situation in the parasite [141].

The genes coding for two *P. falciparum *aminopeptidases were chemically synthesized using codons optimized for the yeast *Pichia pastoris*, but expression in yeast was unsuccessful. In contrast, when these constructs were transfected into *Sf*9 insect cells, soluble recombinant proteins that were enzymatically active were produced in sufficient quantities to perform kinetic and biochemical analyses [142,143]. A subtilisin-like protease (*Pf*SUB-1), involved in merozoite invasion, was expressed and secreted in a baculovirus system and used in functional studies to assess the interaction with its cognate propeptide [144]. A micronemal protein present in *Plasmodium berghei *ookinetes (von Willebrand factor A domain-related protein, *Pb*WARP) was produced in a baculovirus system and retained its function and oligomeric structure [145]. This protein plays a role in the motility and attachment of the ookinete in the mosquito midgut and homologues of this protein exist in *P. falciparum *and *P. vivax *[145].

An important aspect of the baculovirus/insect cell system is that it recognizes eukaryotic targeting signals, which allows expression and processing of different classes of proteins, including those that are secreted, localized to the plasma membrane or nucleus, and cytoplasmic proteins [135,146]. The host insect cell can also perform most post-translational modifications, such as phosphorylation, acylation and glycosylation. The high expression levels of recombinant proteins in the late stages of the viral life cycle can however lead to incomplete post-translational processing, resulting in, for example, hypophosphorylation of some proteins. Glycosylation can also be problematic [132], especially since N- and O-glycosylation only seem to occur at very low levels in *P falciparum *[147]. Native *Pf*EBA-175 is essentially unglycosylated, despite the presence of putative sites, whereas 20% of the recombinant baculovirus protein was N-glycosylated, although this did not affect its immunogenicity [139]. The major carbohydrate modification in *Plasmodium *is the addition of a glycosylphosphatidylinositol (GPI) anchor to the C-terminal amino acid of a protein [147]. GPI-anchored proteins play an important role in numerous cellular functions and are essential for parasite survival. Baculovirus may not be ideal for expressing GPI-anchors since recombinant CSP was devoid of the GPI moiety, despite the presence of a potential GPI cleavage/attachment site in the DNA.

A modification of the baculovirus system is to use a virus specific for silkworm larvae, as opposed to cultured insect cells. The yield of biologically and immunologically active *Pf*MSP-1_42 _recombinant protein from these larvae was > 100-fold higher than from *Sf *cells [148]. However, expertise in handling silkworm larvae is currently centred mainly in China.

**Figure 1:**
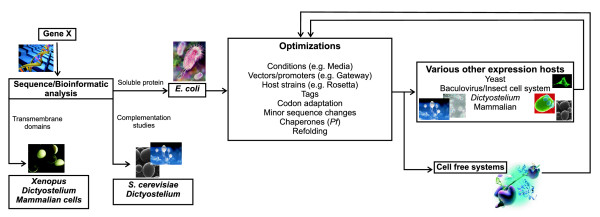
**Generalized strategy for the successful expression of plasmodial proteins.** Extensive sequence analysis of a gene could facilitate genetic manipulation of the gene if needed. *E. coli *should remain the starting point for the expression of soluble proteins due to its ease of use and *Xenopus *remains the preferred choice for membrane protein expression. Several optimizations including codon adaptation, plasmodial chaperone co-expression and small sequence changes might be particularly necessary for the expression of the plasmodial protein of interest. Various other expression hosts including mammalian, baculovirus, yeasts, *Toxoplasma*, *Dictyostelium *and cell free systems have been used with various levels of success and might include an iterative process incorporating necessary optimizations.

The baculovirus system is a valuable tool for the expression of *Plasmodium *proteins and represents a viable alternative to explore, especially since it is difficult to predict which heterologous host will be optimal for a specific protein and also because a universally applicable method to express recombinant malaria proteins is not available (Figure 1). In addition, the system lends itself to automation and scaling up of protein production. However, it requires a considerable investment in time and financial resources and it is technically more demanding than manipulating *E. coli*.

#### *Dictyostelium discoideum*

The single-celled amoeba *Dictyostelium discoideum *has become an important and accessible model organism for studying numerous aspects of eukaryotic cell biology, such as cell division, locomotion, signal transduction and vesicular trafficking, [149]. From the point of view of plasmodial expression, *D. discoideum *offers several attractive features: 1) The relative ease with which it can be cultured and manipulated genetically may be second only to yeast amongst eukaryotic systems, hence its popularity as a model organism [149]; 2) It contains the canonical organelles and cell biological features of eukaryotic cells, and could be used to infer the functional attributes of heterologously expressed plasmodial proteins by their interaction with endogenous proteins. It may be particularly useful for integral membrane protein expression. For example, expression and targeting of *Pf*CRT to the digestive compartments of *D. discoideum *provided evidence for the role of mutations in this membrane protein in reducing chloroquine (CQ) accumulation and hence mediating CQ resistance in parasites [150]; 3) The relative ease with which endogenous *D. discoideum *genes may be disrupted by homologous recombination or silencing offers the potential to create mutants for use in the functional characterization of heterologous proteins by complementation. For example, adenylate cyclase null mutants were used to demonstrate the properties of *Pf*ACα, an integral membrane protein possibly involved in gametocytogenesis [151]; 4) Plasma membrane anchoring via endogenous signal sequences and GPI-anchoring motifs could be used to study the adhesion properties of malaria proteins, e.g. CSP [152]. Additionally, such recombinant *D. discoideum *strains may be explored as potential live, non-toxic, non-pathogenic "sporozoite-mimicking" vaccines [152]; 5) *Dictyostelium discoideum *originally became an attractive vehicle for heterologous plasmodial protein expression due to the unusual A+T bias of its gene coding sequences: a feature it shares with *P. falciparum *[153]. The organism was first used for the preparative expression of malaria vaccine candidates including *P. falciparum *CSP and, subsequently, the C-terminal portions of CSP from *P. yoelii *and *P. falciparum *and a fragment of MSP1 of *P. vivax *[152,154,155]. Although codon optimization was not performed, it was noted that expression was considerably improved by the N-terminal addition of *D. discoideum *leader sequences or fusion partners. Unfortunately, the paucity of reports on the use of *D. discoideum *for the preparative expression of plasmodial proteins prohibits an assessment of the advantages conferred by the organism's A+T bias and its utility in this regard over the more conventional yeast expression systems. Nonetheless, it is possible that *D. discoideum *is underexplored as an alternative to mammalian cells and *Xenopus *oocytes for the functional expression of plasmodial membrane proteins and transporters (see discussions below).

#### *Toxoplasma gondii*

*Toxoplasma gondii *is an important parasite pathogen in immuno-compromized individuals and during pregnancy. Like malaria parasites, it belongs to the phylum Apicomplexa, with the advantage that it is considerably more susceptible to experimental manipulation by standard molecular genetic approaches [156]. Phylogenetic relatedness notwithstanding, the differences in host cell niche, pathogenesis and life-cycles (in addition to a G+C *vs*. A+T codon bias) makes it debatable to what extent the cell biology and metabolic pathways of *T. gondii *are related to those of the various life-cycle stages of malaria parasites. Nonetheless, *Toxoplasma *and *Plasmodium *share the same mechanism of locomotion as well as the specialized secretory organelles and mechanisms required for host cell invasion [157]. Consequently, *T. gondii *may be a useful host for the heterologous expression and characterization of malaria proteins involved in locomotion, invasion and biogenesis of the specialized secretory organelles (rhoptries, micronemes and dense granules). Per illustration, expression of a dominant negative mutant form of *Pf*Vps4 led to the identification of a multi-vesicular body endosomal compartment involved in rhoptry biogenesis in *Toxoplasma *and suggested the existence of similar organelles and their functions in malaria parasites [158]. A further potential use of *T. gondii *expressing plasmodial sequences is as an attenuated live vaccine. Experiments in Rhesus monkeys and mice have demonstrated the promising utility of *T. gondii *strains expressing *P. knowlesi *and *P. yoelii *CSP in this regard [159,160]. From a preparative point of view, the *P. falciparum *cGMP-dependent protein kinase (*Pf*PKG) was expressed and purified from *T. gondii *to characterize its enzymatic properties and susceptibility to inhibitors [161]. Nonetheless, it is unlikely that *T. gondii *will challenge *E. coli *or yeast systems as a first choice for this type of application.

#### Mammalian cells

To a significant extent, mammalian cells share with *S. cerevisiae *the features of being well characterized, having versatile reagents and protocols for transgene expression and analysis as well as potentially aiding correct folding of heterologous proteins in secretory compartments and imparting post-translational modifications (e.g. lipid modifications, GPI-anchoring, etc.). However, the substantially lower yields of recombinant proteins minimize their use for preparative expression. An advantage of mammalian cells is the ability to target heterologous membrane proteins to subcellular compartments and the cell surface. As a result, the large majority of studies reporting the expression of plasmodial proteins in mammalian cells have focused on the identification and characterization of parasite surface ligands that mediate host cell binding. These experiments have significantly contributed to our understanding of erythrocyte invasion by helping to identify merozoite proteins and their domains that mediate erythrocyte binding, notably EBA-175, AMA-1, Duffy-binding protein, JESEBL, MAEBL, MSA-1 and *Pf*AARP (e.g [162-170]). In addition, the studies have characterized regions of *Pf*EMP-1 involved in binding to host chondroitin sulphate A (CSA), ICAM-1, CD36 and uninfected erythrocytes, thus mediating parasite sequestration to the placenta and vascular endothelium as well as erythrocyte rosetting in infected individuals [171-174]. A concomitant advantage of surface expression has been to map out conserved epitopes recognized by inhibitory monoclonal antibodies and human sera (e.g. [175-177]). To facilitate the surface binding experiments, parasite protein domains are modified by N- and C-terminal fusion to mammalian or viral secretory signal sequences and transmembrane domains/GPI-anchoring motifs, respectively, and tagged with an epitope tag and/or GFP to monitor expression. The COS-7 green monkey kidney cell-line has generally been used for these (and other plasmodial expression) experiments, but the well-known CHO-K1 and HeLa cell lines have also been employed. Interestingly, with few exceptions (e.g. [174,177]), the plasmodial sequences were rarely altered to rectify A+T-codon bias and remove potential N-glycosylation sites.

The second most numerous application of mammalian cell expression in malaria research is to support the development of DNA plasmid-based vaccines. Transfection of mammalian cells with the vaccine plasmids is used to verify expression of the relevant plasmodial sequences prior to proceeding with animal vaccination. In addition, mammalian cell expression may also be used to assess the generation of the desired specific antibodies subsequent to vaccination (e.g. [178-181]). Again, codon optimization is rarely reported. This raises the possibility that mammalian cells may be more accommodating for the expression of the A+T-biased plasmodial sequences.

Despite the advantages offered by the extensive characterization of mammalian cell biology, the cells appear to be rarely used for functional characterization of plasmodial proteins, beyond the ligand binding studies described above. One likely reason is the comprehensive difficulty of preparing functionally compromized mutants, which hampers the complementation studies that have been extensively done in *S. cerevisiae *(and potentially *D. discoideum*). A prospective (unreported) alternative may be to use post-transcriptional silencing (e.g. RNAi-mediated) to generate deficient cells for complementation assays. In addition, provided the functional readout is sufficiently sensitive and robust, overexpression of plasmodial proteins above the endogenous background may yield useful results. A further advantage for functional characterization is the high transient transfection efficiencies that can be achieved with current transfection reagents and cell types (e.g. COS cells), which may increase the turnaround time from transfection to analysis. Examples have included the cytoplasmic expression of CDP-diacylglycerol synthase (*Pf*CDS), mannosyl transferase (*Pf*PIG-M), *Pf*VPS4 and histidine rich protein II HRPII, which has shed light on the potential roles of these parasite proteins in phospholipid synthesis, GPI-anchor synthesis, endocytic trafficking and pH-sensing, respectively [158,182-184].

Both *Plasmodium *membrane transporters implicated in CQ resistance, Pgh1 and *Pf*CRT, have been functionally expressed in mammalian cells. Although the intracellular location of Pgh1 expressed in CHO cells could not be determined accurately, it increased CQ uptake and sensitivity in these cells, whilst Pgh1 incorporating CQ resistance-associated mutations did not [185]. After synthetically introducing a yeast codon bias into the *Pf*CRT cDNA sequence, the protein was expressed in HEK 293 cells and faithfully targeted to lysosomal membranes [186]. Surprisingly, in contrast to the results obtained in *D. discoideum *([150]; see earlier discussion), *Pf*CRT did not alter CQ accumulation in the host cells but increased acidification of lysosomes, an effect which was enhanced when the CQ resistance-conferring mutation K76T was included in the protein. The latter contradiction highlights the possibility that conclusions drawn from characterization in heterologous cell types may not always translate to accurate functional prediction in the parasite and does not substitute for characterization in the parasite itself.

#### *Xenopus laevis*

Once the technical and logistical challenges of *Xenopus *oocyte micro-manipulation and cRNA injection have been overcome, the cells may be extremely useful for the functional expression and characterization of membrane proteins, notably transport proteins. It has become the method of choice for characterizing *Plasmodium *transporters, particularly since the oocytes appear to be more tolerant than other eukaryotic cells to the peculiar codon preferences of *Plasmodium *genes [187,188]. The expression system has been used most extensively to characterize *Pf*HT1, the principal hexose transporter of *P. falciparum *[188], in order to define its substrate specificity, probe structure-function relationships by mutational analysis with a view to therapeutic inhibitor design and confirm it as a potential drug target [189-191]. The substrate specificity of *Pf*ENT1, one of the four equilibrative nucleoside transporters encoded by the *P. falciparum *genome, was also determined using *Xenopus *oocyte expression and compared favourably with results obtained from transport experiments using isolated parasites, thus validating the oocyte system as a more convenient surrogate for studying this transporter [192-195]. A similar comparison of properties between isolated parasites and heterologous expression in *Xenopus *oocytes was obtained for Na^+^- and membrane potential-dependent transport of inorganic monovalent phosphate by *Pf*PiT [196]. Additional transporters characterized by expression in *Xenopus *oocytes coupled with oocyte swelling assays include the aquaglyceroporins of *P. falciparum *and *P. berghei *(*Pf*AQP and *Pb*AQP) [197,198].

A significant contribution of the *Xenopus *oocyte expression system to malaria drug discovery was the identification and characterization of the foremost target of the artemisinins, *Pf*ATP6, a SERCA-type Ca^2+ ^ATPase [199]. The study followed on the characterization of *Pf*ATP4, a *P. falciparum *P-type membrane Ca^2+^-ATPase, *vis-à-vis *its susceptibility to inhibitors and Ca^2+ ^concentration-dependent activation [200]. Of further relevance to drug discovery was the use of *Xenopus *oocyte expression to investigate the role of *Pf*CRT in conferring CQ resistance. The findings led the authors to propose that *Pf*CRT affects CQ susceptibility in parasites by a modulation of food vacuole transporters, rather than by directly affecting vacuole pH or transporting CQ [201].

It should be noted that, although the *Xenopus *oocyte expression system has been used almost exclusively to characterize malaria membrane transporters, it is a functional cell and can be a useful system for studying other cellular functions as well, for example, cell signalling. After expression in oocytes, the parasite leucine-rich repeat antigen (*Pf*LRR1) was found to bind to the endogenous *Xenopus *protein phosphatase PP1 and disrupt the oocyte G2/M cell cycle arrest, observed as germinal vesicle breakdown. By inference, *Pf*LRR1 probably regulates protein phosphatase activity and cell cycle progression in malaria parasites [202]. In addition, measurement of cAMP levels in oocytes was used to characterize the properties of a *X. laevis *codon-optimized version of the parasite adenylyl cyclase (*Pf*ACα) and confirm results obtained by complementation assays in *Dictyostelium *[151].

#### Plants

The use of plants as expression hosts for foreign proteins has received increasing attention. Several heterologous proteins have been expressed in plants due to their ability to successfully express, fold and post-translationally modify foreign proteins. The real potential lies in the possibility of transgenic plants as bioreactors for the production of vaccines since the feasibility and low cost have been validated in several human and animal disease vaccines. It is therefore attractive to speculate that plants may be a host for 1) the transient production of functional plasmodial proteins or 2) the transgenic production of e.g. malaria vaccine targets. The latter has been under investigation with reports by Ghosh *et al*. (2002) in which they successfully obtained constitutive expression of a 19 kDa proteolytic product of *P. falciparum *MSP1 in tobacco plants resulting in the same immunogenic properties as an *E. coli *expressed form of the protein [203]. More recently, the concept of edible vaccines derived from antigen production in plants was supported by evidence from Hepatitis-B trials in humans and mice and has now been extended to the *P. yoelii *MSP4/5 expressed in transgenic tobacco [204]. This protein was codon optimized for production in plants and resulted in the high level expression of the antigen that could elicit protective antibodies in mice fed orally. These reports therefore indicate the feasibility of exploiting plants for the production of malaria proteins.

#### Cell-free protein expression

Cell-free transcription-translation systems have been exploited with varying degrees of success for numerous proteins including membrane proteins and cytotoxic proteins (reviewed in [205,206]). Advantages include production of proteins that undergo proteolysis or that usually accumulate in inclusion bodies, as well as allowing selective labelling. Of recent interest is the wheat germ cell-free platform [206], which allows the high-level expression of eukaryotic proteins that can not be expressed in *E. coli *cells/cell-free systems. Optimized wheat germ systems have been created by removal of ribosomal protein synthesis inhibitors (tritin) from the wheat embryo to allow the robust production of proteins. This technology has been adopted by structural genomics consortia (e.g. Centre for Eukaryotic Structural Genomics, Madison, WI). The difficulties experienced over many years in obtaining significant soluble expression of *Pf*DHFR-TS are well-documented [207]. In a recent study it was shown that a wheat germ cell-free expression system yielded bioactive protein as an obligate dimer without the associated toxicity observed in cell-based systems [207]. Moreover, several malaria vaccine targets (*Pfs*25, *Pf*CSP and *Pf*AMA-1) were successfully expressed in both native and codon-optimized forms which did not seem to influence the efficiency of expression, at least of *Pfs*25 [208]. Yields between 50 and 200 μg/ml of soluble protein were obtained which was subsequently shown to be immunogenically active in raising specific antibodies in mice. Ambitiously, this study was expanded to include 124 additional *P. falciparum *blood stage proteins that were expressed in their native forms. Of these, 75% of the proteins were produced with an average yield of 1.9 μg/150 μl reaction mixture and 65% solubility. Inverse correlations were observed between soluble protein production and protein size, frequency of low-complexity regions and pI of the proteins.

## Conclusion and future perspectives

Heterologous expression of plasmodial proteins as described above is principally carried out for preparative purposes, for example, crystallization and structural studies, preparation of recombinant vaccines, drug screening, antigen preparation, enzymatic and other *in vitro *functional assays, etc. However, in light of our poor understanding of parasite cell biology and the fact that the majority of malaria proteins predicted by the genome have unknown or uncertain functions, an important concomitant activity is the elucidation of *Plasmodium in vivo *protein function by heterologous expression in experimentally tractable, well-characterized eukaryotic model organisms and cell types. Model eukaryotes are especially favoured for the expression and characterization of integral membrane proteins, for which prokaryotes are generally ill-suited, provided that spurious, incorrect post-translational modifications (notably glycosylation) in the secretory pathway of the host cell can be avoided or tolerated. In addition to *in situ *functional characterization of malaria proteins, eukaryotic cells have also been explored for preparative expression in an attempt to circumvent problems experienced in prokaryotes, notably incorrect protein folding, insolubility and codon incompatibility, as described in the preceding sections.

From the above, it is evident that each protein has to be assessed on an individual basis since there is no universal procedure for the heterologous expression of malaria proteins or proteins of other organisms. However, a protocol is suggested worthy of consideration when attempting to express a plasmodial protein (Figure 1). Bioinformatics analyses of the gene and its encoded protein is clearly of paramount importance to guide the selection of the appropriate gene construct, the host/vector combination and the expression and isolation procedures. Expression of proteins from *E. coli *systems is clearly the first choice due to its ease of genetic manipulation and the availability of a variety of different vectors and *E. coli *strains. Fortuitously, new strains, vectors and methods are continuously being developed to address the multitude of challenging impediments revealed by structural genomics consortia in their endeavours to determine the three-dimensional structures of proteins encoded by the genomes of Eubacteria, Archea and Eukarya.

Alternative hosts including yeasts, baculovirus and cell-free systems, with or without codon adaptation strategies, are additional options to be explored if expression from *E. coli *systems is problematic. For preparative expression of soluble proteins, *Pichia pastoris*, baculovirus and cell free systems may be the most successful whereas alternatives such as *Saccharomyces, Dictyostelium *and mammalian systems may be more beneficial for functional analyses with the first two additionally having the major advantage of complementation studies thereby circumventing soluble protein isolation. Attempts to express transmembrane proteins for functional analyses should be focussed on systems including Xenopus and mammalian systems with a potential application of *Dictyostelium *as host.

The introduction and ongoing development of reverse genetics methodology for *P. falciparum *[e.g[209]], coupled with sporadic reports of successful post-transcriptional silencing by antisense RNA, RNAi and ribozymes [e.g. [210,211]], provides the primary tools for elucidating protein function in the parasite. Nonetheless, transgene expression in more tractable heterologous model organisms (notably through mutant complementation in yeast and surface expression in *Xenopus *oocytes and mammalian cells) offers a powerful parallel approach to aid functional characterization. These transgene expression strategies are especially useful for determining substrate or ligand specificity, structure-function relationships through mutational analysis, and inhibitor sensitivity/resistance, and, under optimal circumstances, may even replace the requirement for preparative expression of purified soluble protein.

It is clear that numerous options are available, and will continue to be developed, for the successful expression of plasmodial proteins. This facet of research, however, still represents a major challenge to scientists in the malaria field, but it is a vital first step in the post-genomic functional and structural characterization of these proteins. This knowledge is crucial to identify new proteins that may serve as potential drug or vaccine targets, which will enhance our chances of producing new therapies to combat malaria in the future.

## Competing interests

The authors declare that they have no competing interests.

## Authors' contributions

The South African Malaria Initiative  was initiated with the purpose of integrating malaria research expertise in South Africa to accelerate novel drug and drug target discovery. All authors are members of the Functional Expression of malaria proteins Core Expertise Group of SAMI. AIL conceptualized the manuscript; all authors were involved in drafting the manuscript, particularly LB, EH who were also responsible for the codon harmonization, protein refolding, plant and cell free sections; GB, AS and LS were responsible for the chaperone section; TLC for the baculovirus section; HH for the *Dictyostelium*, *Toxoplasma*, mammalian cell and *Xenopus *sections; LO contributed to the yeast section; ZN coordinates the SAMI research effort; EJM chairs the SAMI steering committee.
